# Supplementary material for: Top-down feedback controls spatial summation and response amplitude in primate visual cortex
Source: Nat Commun. 2018 Jun 11;9:2281. doi: 10.1038/s41467-018-04500-5 (PMC5995810; doi:10.1038/s41467-018-04500-5)
Supplement: Supplementary file 1 — Supplementary Information [file 41467_2018_4500_MOESM1_ESM.pdf]

SUPPLEMENTARY INFORMATION

SUPPLEMENTARY NOTES

Supplementary Note 1: Analysis of RF Alignment

For each array penetration, we quantified the alignment of the minimum response fields (mRFs) mapped at each contact across the array. For each contact, we calculated the Euclidean distance of the mRF center, mapped at that contact, from the mean of mRF centers in that penetration. For the purpose of this analysis, the mRF center was defined as the center of mass of the thresholded mRF activity map (responses >1SD above baseline; **Supplementary Fig. 1b**). The median Euclidean distance from the mean mRF center of each penetration was 0.124º±0.029º/0.035º (95% CI lower bound/upper bound; bootstrap).

Supplementary Note 2: Control Experiments in Cortex Not Expressing ArchT

For the main experiment, laser intensities were selected based on a control experiment in one animal (n=2 penetration) in cortex not expressing ArchT. Recordings and analysis were otherwise identical to the main experiment. Unsorted, thresholded multi-units were analyzed for this control.

We found light artifacts at relatively low light intensities (63mW/mm^2; see **Supplementary Fig. 2a**), which, to our surprise, have been used in previous optogenetic experiments. The laser artifacts were qualitatively different in superficial and deep layers: spike-rates were usually increased in superficial layers, but often decreased in deep layers (**Supplementary Fig. 2a)**. For granular and infragranular layers, irradiances at or below 43mW/mm^2 did not produce statistically significant changes in the cells' size tuning curves (mean sRF size±s.e.m no-laser vs. laser: granular 1.84±0.04º vs. 1.76±0.11º p=0.60, n=5; infragranular: 2.75±0.75º vs. 2.50±1.0º, p=0.91, n=2; **Supplementary Fig. 2a-b**). For some contacts in supragranular layers, instead, the laser-on and control curves differed significantly at 43mW/mm^2 irradiance. Importantly, however, the effect of light on these cells was always a *decrease* in sRF diameter, i.e. an effect opposite to that caused by the laser in ArchT expressing cortex (supragranular: 1.57±0.11º vs. 1.24±0.09º p=0.006, n=14 **Supplementary Fig. 2a-b)**. These laser-induced artifacts in supragranular cells disappeared at lower laser intensities. Specifically, when we repeated the control sRF size analysis including supragranular units at laser irradiances of <43mW/mm^2, (specifically 19mW/mm^2; n=12 units for this analysis as we did not have recordings at 19mW/mm^2 laser irradiance for all units), and granular and infragranular contacts at laser irradiance of 43mW/mm^2, we found no statistically significant changes in sRF diameter (mean±s.e.m no-laser vs. laser: 2.1±0.20º vs. 1.8±0.18º, p=0.36, n=12, mean decrease 6.68±6.96%; **Supplementary Fig. 2c**) or response amplitude in the proximal surround (no-laser vs. laser: 60.9±12.3 vs. 59.6±13.8 spikes/s, p=0.72, n=12, mean decrease 7.7±8.30%; **Supplementary Fig. 2d**).

The analysis reported in the Results (**Figs. 2-3**) was performed for laser intensities up to 43mW/mm^2, because the laser artifacts induced in some supragranular cells at this intensity could not account for the observed effects of feedback inactivation (as these artifacts caused

decreases rather than increases in sRF size). However, to further corroborate that our results of feedback inactivation could not be attributed to laser-induced artifacts, we repeated all the main analyses of data recorded in ArchT-expressing cortex, after excluding supragranular units which showed inactivation effects at laser irradiances >19mW/mm^2, i.e. irradiance levels that had produced artifacts in some supragranular layer cells in control cortex. The results of this analysis were qualitatively and quantitatively similar to those of the original analysis, as detailed below.

Analysis of Data in Cortex Expressing ArchT, Excluding Supragranular Cells Showing Inactivation Effects at >19mW/mm^2 Irradiance.

Mean sRF diameter was significantly smaller with intact feedback, compared to when feedback was inactivated (mean±s.e.m no-laser vs. laser: 1.24±0.11° vs. 1.83±0.17°, p=0.007, n=26; **Supplementary Fig. 3**), with a mean increase of 59.3±13.0% (p<0.001). As for the original analysis (**Fig. 3a-c**), stimuli extending into the proximal surround evoked larger neuronal responses (no-laser vs. laser: 42.0±15.4 vs. 51.8±21.5 spikes/s, mean increase 30.0±6.34%, p<0.01; **Supplementary Fig. 3b**), and therefore less surround suppression, when feedback was inactivated compared to when feedback was intact. Laser stimulation reduced the suppression index (SI) for stimuli covering the sRF and proximal surround (SI no-laser vs. laser: 0.18±0.03 vs. -0.02±0.06, p<0.01; **Supplementary Fig. 3c**). In contrast, the response (no-laser vs. laser: 13.1±2.63 vs. 13.3 ±2.73 spikes/s, mean spike-rate increase 10.6±11.2%, p=0.37) and SI (no-laser vs. laser: 0.57±0.05 vs. 0.55±0.06; **Supplementary Fig. 3d**) evoked by stimuli extending into the more distal surround were unchanged by feedback inactivation. Stimuli confined to the neurons' sRF evoked lower responses in the laser condition (41.1±19.2 spikes/s) vs. the non-laser condition (50.1±17.6 spikes/s, mean reduction 30.3±6.34%, p<0.001; **Supplementary Fig. 3e**). We conclude that increased sRF diameter and reduced surround suppression indeed resulted from inactivating V2 feedback to V1, and were not caused by laser-induced heat artifacts.

None of the units recorded in the control experiment showed reduced response at the irradiances used for the analysis of data in **Fig. 4**. Thus, we are confident that the general response suppression for small and large stimuli observed in the data reported in **Fig. 4** resulted from inactivating feedback axons.

Supplementary Note 3: Analysis of sRF Size Increase Induced by Noise

We performed an analysis to exclude the possibility that the increased sRF size after feedback inactivation could arise from noise in the spatial summation data. For each cell, we first generated a size-tuning curve from the fitted ROG or DOG model, depending on which model better fitted the cell's response. For each cell, we then generated a "noisy" curve by independently sampling for each presented stimulus diameter 10 responses from a Poisson distribution having the same mean as the "real" fitted curve at those diameters. These 10 sampled responses per stimulus diameter were then averaged to produce a noisy version of the real curve (**Supplementary Fig. 4a**), and the peak of the noisy curve was taken as the sRF size. This procedure was repeated 10,000 times for each cell. For each repetition, we computed the percent increase in sRF size between the real curve and the noisy curve for the cell, as done for the recorded data. Finally, we produced a distribution of the average percent increase in sRF size across the population of cells in this simulation. The median increase in sRF size in the bootstrapped distribution was 3.6%, as opposed to the 56% increase seen in the real data

(**Supplementary Fig. 4b**). We thus conclude that feedback inactivation increases sRF size significantly more than would be expected from noise.

Supplementary Note 4: Analysis of Surround Size

For the population of cells showing an increase in sRF diameter when feedback was inactivated, we found no changes in the size of the surround field (see Methods for definition). Average surround diameter in the no-laser vs. laser condition was 4.71±0.43º vs. 5.38±2.77º ($p=0.33$).

Supplementary Note 5: Control Analysis for Laser Stimulation Time

Inactivation of axon terminals using ArchT can, counter intuitively, facilitate synaptic transmission for prolonged light pulses, while ArchT is consistently suppressive for pulse widths of ≤ 200ms. Thus, we repeated our analysis by focusing only on the first 200ms of the response. We found no qualitative differences between the original analysis and the short time-scale analysis. Consistent with the original analysis, sRF diameter was increased when feedback was inactivated (no-laser vs. laser: 1.14±0.07º vs. 1.67±0.24, $p<0.05$, $n=19$ units producing reliable responses within the initial 200ms), responses to stimuli confined to the sRF were significantly reduced (no-laser vs. laser: 26.1±8.89 vs. 21.6±10.3 spikes/s, mean spike-rate reduction 45.1±8.62%, $p<0.001$), and responses to stimuli covering the sRF and proximal surround were increased (mean spike-rate increase 67.6±34.0%, $p<0.06$). We conclude that the observed laser-induced effects reflect suppressed, rather than facilitated, V2 feedback activity.

Supplementary Note 6: Phenomenological Modeling

To gain insights into the mechanisms underlying feedback inactivation effects, we fitted ROG and DOG models to the data (see Methods) and determined which model parameters were mostly affected by feedback inactivation. To this goal, we selected for each cell the model that provided the best fit to its size tuning measurements (based on the averaged R^2 over laser and no-laser conditions) in the no-laser condition, and then allowed one parameter at a time to vary with feedback inactivation, while the remainder of the model parameters were held fixed to their original values for the no-laser condition. The model in which feedback inactivation modified the spatial extent of the center excitation (w_c in equations 1-3) provided the best fit for 45% of the cells (top left panel in **Supplementary Fig. 5a**). This model could capture the increase in sRF size and response reduction for small stimuli, seen in the data after feedback inactivation, but failed to capture increases in response for stimuli extending into the surround. In contrast, a model in which feedback inactivation modified the spatial extent of the surround inhibition (w_s in equations 1,2,4) could capture changes in response for stimuli extending into the surround, but failed to capture changes in sRF size (top right panel in **Supplementary Fig. 5a**). Moreover, none of the single parameter models could capture the co-occurrence of response reduction to small stimuli and response increase in the proximal surround often seen in the data (**Supplementary Fig. 5a**). We next allowed two parameters at a time to vary with feedback inactivation, while holding the rest constant, and performed this analysis for all possible combinations of parameter pairs. A model in which the spatial extent and gain of the center

excitatory mechanism (w_c and g_c, respectively, in equations 1-3) were simultaneously varied provided the best fit for the largest proportion (30%) of cells (**Supplementary Fig. 5b**). A model in which the spatial extent of both the excitatory (w_c) and inhibitory (w_S in equations 1,2,4) mechanisms were varied performed second best, providing the best fit for 21% of cells. For the model in which w_c and g_c were free parameters, the w_c parameter, describing the spatial extent of the center excitation, typically increased as a result of feedback inactivation, paralleling the sRF size increase seen in the data (**Supplementary Fig. 5c**). This model, was also able to account for a variety changes in response amplitude, as seen in the data, including co-occurrence of reduced response for stimuli inside the sRF and increased response for stimuli in the proximal surround (*solid green curve* in the inset of **Supplementary Fig. 5b**), as well as overall reduced response throughout the entire spatial summation curve (*dashed green curve* in inset of **Supplementary Fig. 5b**). The model in which w_c and w_s were free parameters could also account for co-occurrence of reduced response for stimuli inside the sRF and increased response for stimuli in the proximal surround. Previously, a model in which the gain of the center and surround mechanisms (g_c and g_s, respectively) were free parameters was found to provide a good description of contrast-dependent changes in sRF size[1], which resemble the effects of feedback inactivation we have observed in some cells, especially at high laser intensity. Such a model provided the best fit for only 2 cells in our population (**Supplementary Fig. 5b**), as it failed to account, at least in a straightforward way, for the co-occurrence of response reduction to stimuli in the sRF and response increase in the proximal surround, often seen in the data.

However, we found that the different models performed similarly when compared based on the coefficient of determination (R^2) distributions, rather than fraction of cells best fit by each model (**Supplementary Fig. 5d**).

SUPPLEMENTARY REFERENCES

1. Cavanaugh, J. R., Bair, W. & Movshon, J. A. Nature and interaction of signals from the receptive field center and surround in macaque V1 neurons. *J. Neurophysiol.* **88**, 2530-2546 (2002).

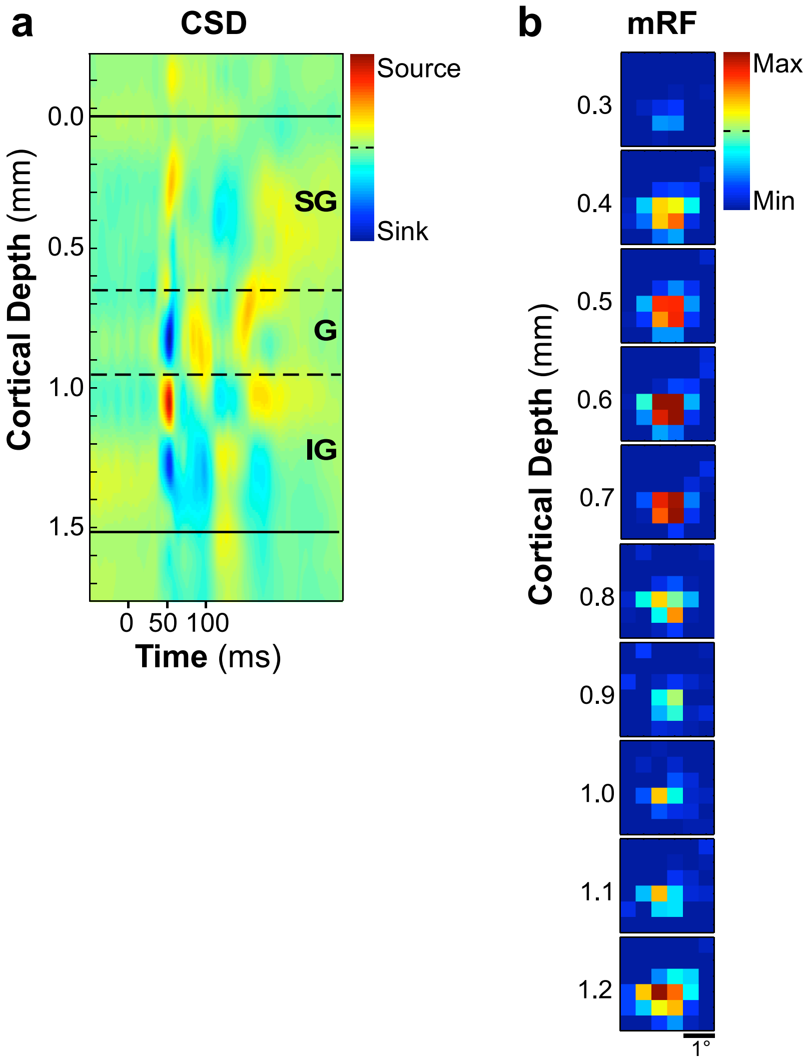

Supplementary Figure 1. Recordings of CSD and minimum RF (mRF) ensure linear array spans all cortical layers, and is positioned normal to the cortical surface.

(a) Current source density (CSD) analysis of local field potential (LFP), used to determine cortical layers and ensure contacts span the full extent of the cortical sheet. **(b)** mRF mapping (see Methods) across contacts through the depth of a single array penetration in V1. Hot spots (regions of max spiking rate) are aligned across contacts, confirming the array is positioned normal to the V1 surface. We also quantified the mRF alignment across all penetrations (see Supplementary Notes, *Analysis of RF alignment*).
SG: Supragranular layers, *G*: Granular layer, *IG*: Infragranular layers.

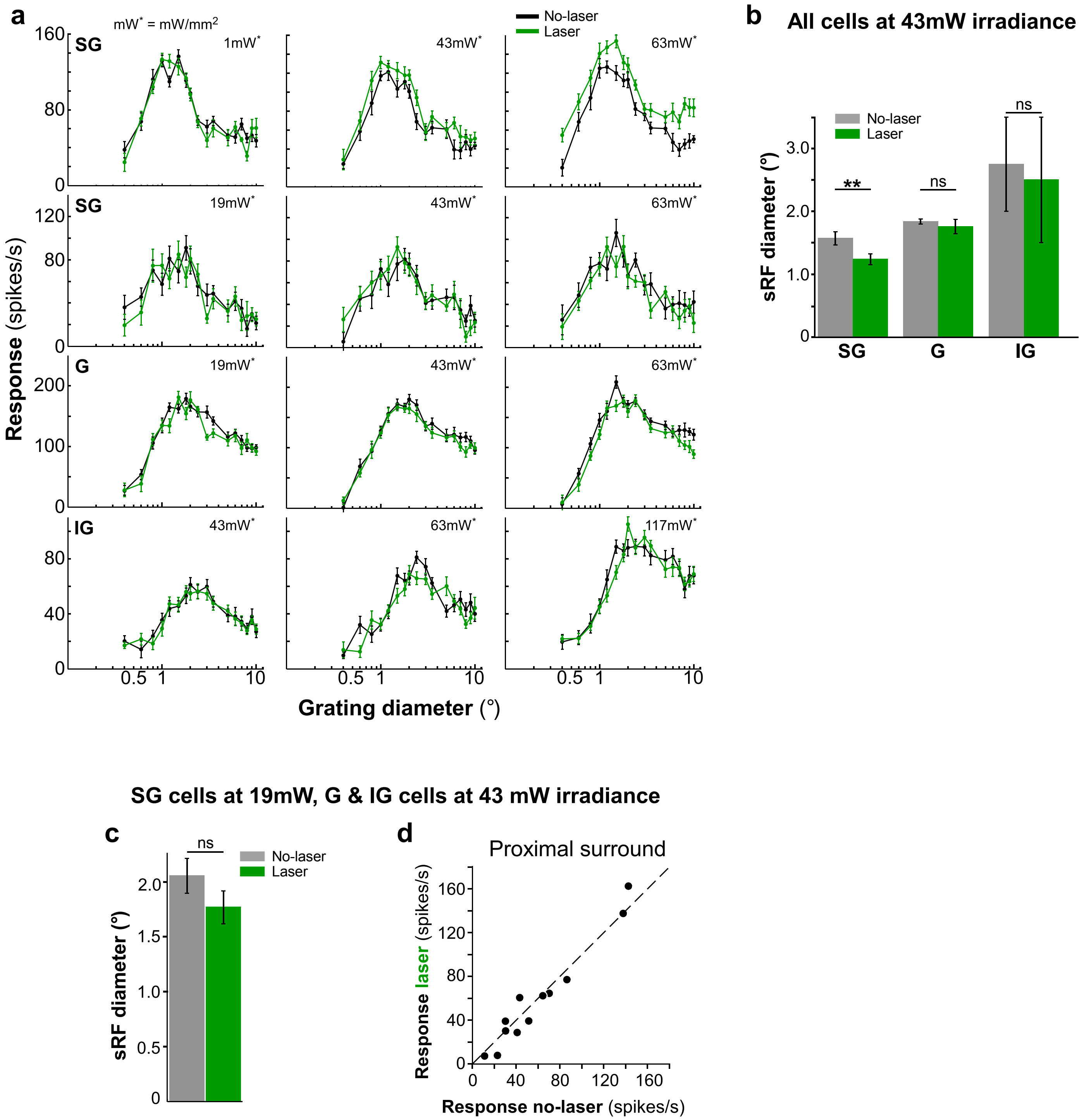

Supplementary Figure 2. Analysis in control cortex not expressing ArchT.

(a) Spatial summation curves for two supragranular (SG), a granular (G), and an infragranular (IG) example cells with (*green*) and without (*black*) laser stimulation at different light intensities (irradiance indicated). **(b)** Mean sRF diameter, with and without laser stimulation, for the whole population of multiunits at laser irradiance of 43mW/mm^2, grouped by layer location. **(c)** Mean sRF diameter, with and without laser stimulation, for the whole population of cells including SG cells treated at laser irradiance of 19mW/mm^2 and G and IG cells at laser irradiance of 43mW/mm^2. **(d)** Response with and without laser for stimuli involving the sRF and proximal surround, for the same population of cells as in (c).

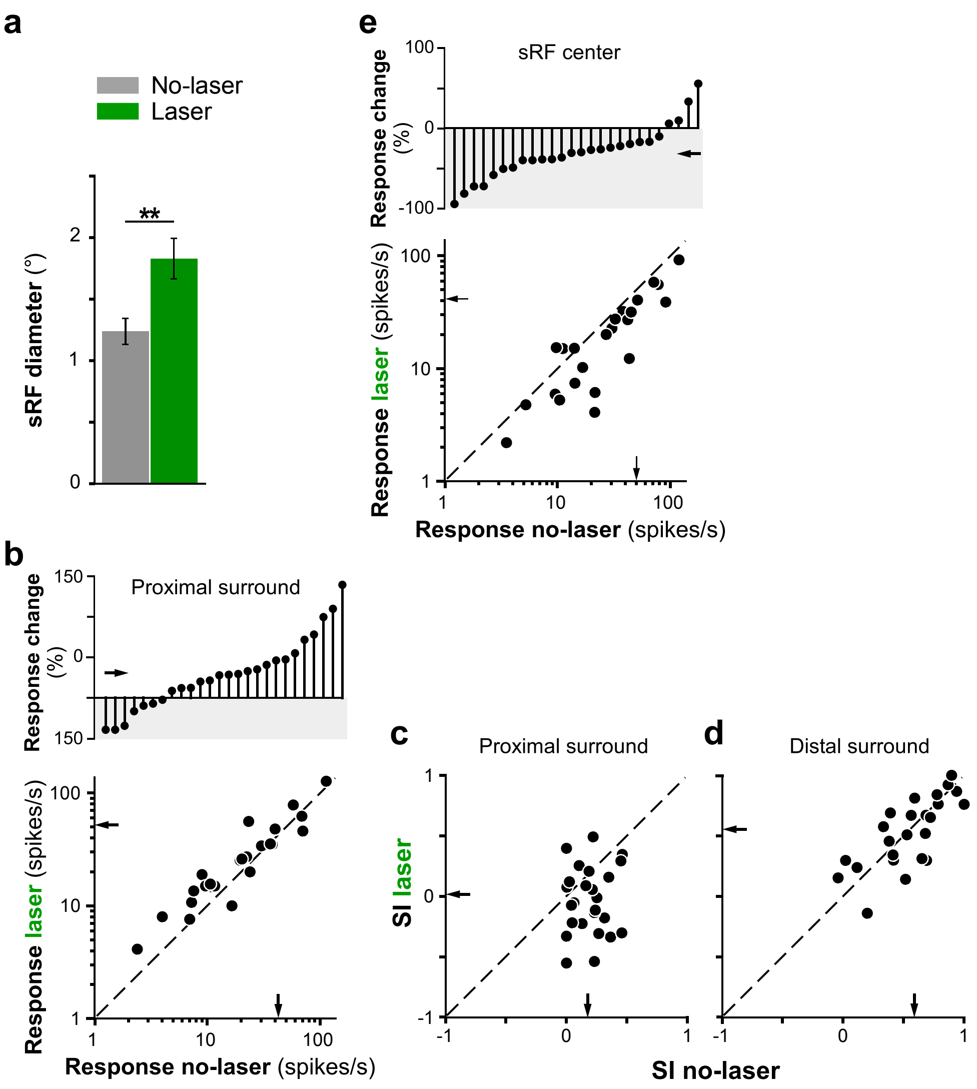

Supplementary Figure 3. Analysis in cortex expressing ArchT, excluding supragranular cells showing inactivation effects at laser irradiance >19 mW/mm^2.

(a) Mean sRF diameter with and without laser stimulation. **(b-d)** Changes in surround suppression with V2 feedback inactivated. **(b)** BOTTOM: response with and without laser for stimuli involving the sRF and proximal surround. TOP: Cell-by-cell percent response change induced by laser stimulation, for stimuli extending into the proximal surround. Upward stems: increase in response with laser stimulation; downward stems (*gray shading*): decrease in response with laser stimulation. **(c)** SI with and without laser for stimuli extending into the proximal surround. **(d)** Same as (c) but for stimuli extending into the distal surround. **(e)** BOTTOM: response with and without laser for stimuli confined to the sRF. TOP: Cell-by-cell percent response change induced by laser stimulation, for stimuli confined to the sRF. *Arrows in (b-e):* means.

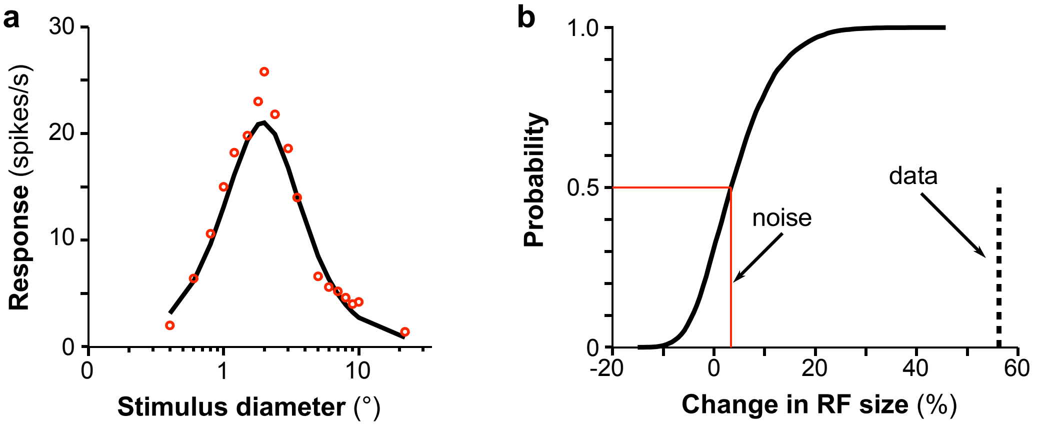

Supplementary Figure 4. Analysis of sRF size increase induced by noise.

(a) The *black curve* represents the “real” size tuning curve derived from phenomenological model fits to the spatial summation data of an example V1 cell. The *red circles* represent the simulated response at each stimulus diameter averaged over 10 trials. The response in each trial was obtained by randomly sampling from a Poisson distribution with the same mean as in the “real” curve. **(b)** Cumulative distribution of percent sRF size changes expected under the null hypothesis that the real size tuning curve measured with and without laser stimulation were identical, and all changes in sRF size were due to noise. sRF size change due to noise was computed separately for each cell, based on a “noisy” tuning curve formed by averaging simulated responses in 10 trials, then averaging over the population of 33 cells and repeating the procedure 10,000 times (for details see Supplementary Notes, *Analysis of sRF Size Increase Induced by Noise*).

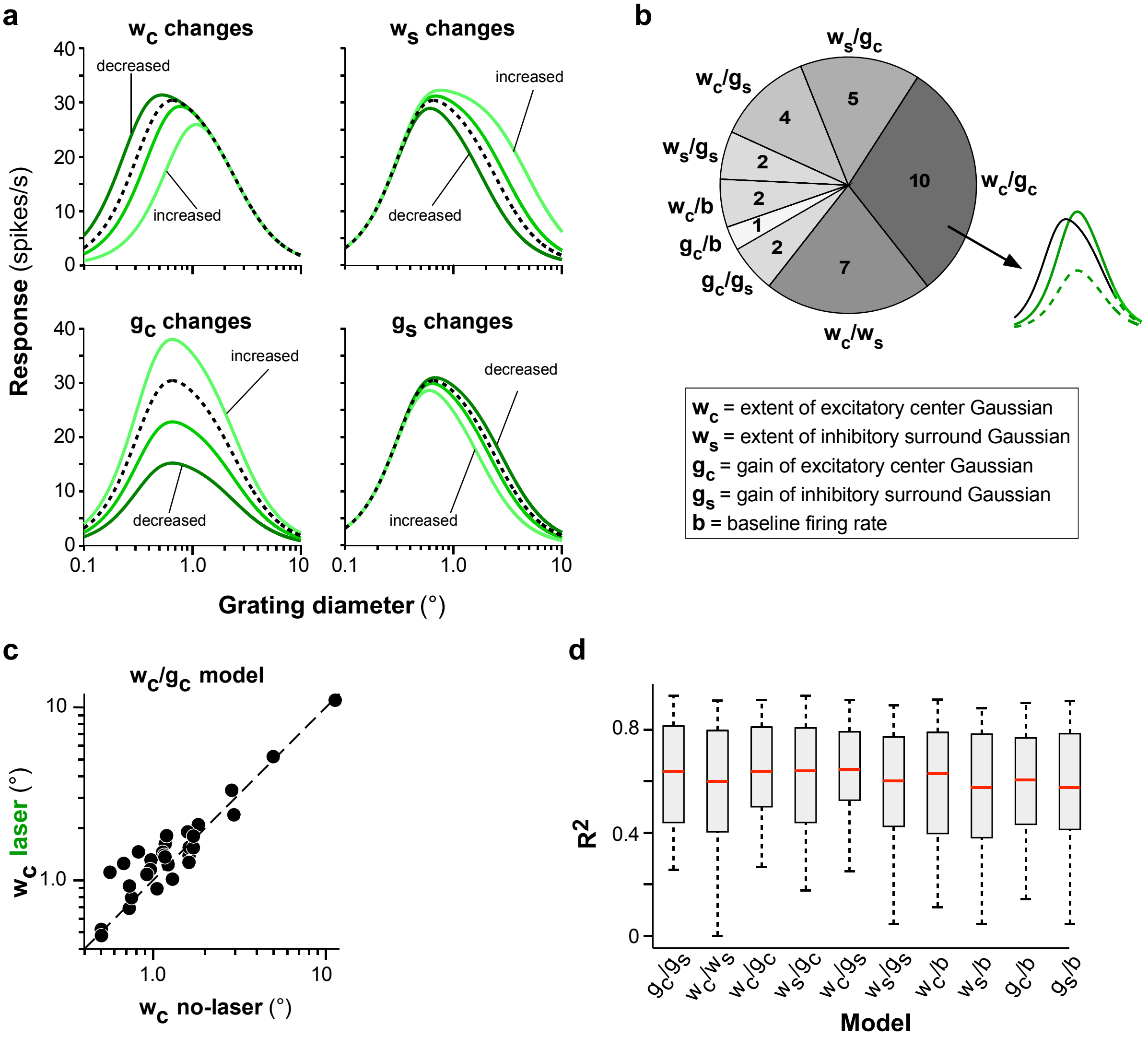

Supplementary Figure 5. Phenomenological models.

(a) Effects of feedback inactivation for each of 4 different versions of the phenomenological models (DOG and ROG), in which only one parameter at a time was allowed to vary while keeping the other parameters fixed to their value in the model that provided the best fit to size tuning measurements in the no-laser condition (*black curve*). In each panel, the *black curve* represents the best fit to the spatial summation data in the no-laser condition of the same representative V1 cell; the *green curves* represent changes in the summation curve when only one parameter (the one indicated at the top of the panel) was allowed to change while keeping the others fixed at their values in the black curve. Different shadings of green indicate different levels of feedback activity, achieved in the model by scaling the free parameter by 0.75 (*darkest green*), 1.25 or 2 (*lightest green*), in all panels, but the top left, for which scaling factors of w_c were 0.5, 0.75 and 1.25, respectively. None of the models could account for the co-occurrence of sRF size increase and increased response in the proximal surround, as often seen in the data (see **Fig. 2a**). **(b)** Proportion of cells for wich each of the two-parameter models (in which only two parameters at a time, i.e. those indicated next to each wedge, were allowed to vary) provided the best fit to the inactivation data. *Numbers* in each wedge indicate the number of cells best fitted by each model. The *inset* indicates the two main effects of feedback inactivation on size tuning that the w_c/g_c model could account for. **(c)** Scatterplot of the spatial extent of the center Gaussian mechanism (w_c) in the no-laser vs. the laser condition, in the w_c/g_c model. Feedback inactivation in this model generally increased the spatial extent of the center Gaussian. **(d)** Distribution of R^2 for each of the two-parameter models. *Red lines*: medians.
